# Supplementary material for: Causal Relationship between Aging and Anorexia Nervosa: A White-Matter-Microstructure-Mediated Mendelian Randomization Analysis
Source: Biomedicines. 2024 Aug 16;12(8):1874. doi: 10.3390/biomedicines12081874 (PMC11351342; doi:10.3390/biomedicines12081874)
Supplement: Supplementary file 1 [file biomedicines-12-01874-s001.zip › Supplementary Figure/Figure S8/Leaveoneout plot of MR analysis for TL to ubm-b-1657.pdf]

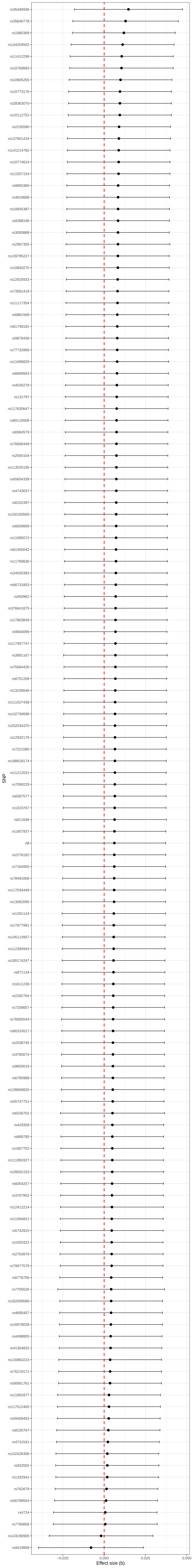

rs35446936
rs35640778
rs1985369
rs144204502
rs11412296
rs10768683
rs10905255
rs10773176
rs28363070
rs10112752
rs2230590
rs137901416
rs141214782
rs10774624
rs11557154
rs9955360
rs4616688
rs10845387
rs9398196
rs3093888
rs2967355
rs139795227
rs10840270
rs12925933
rs73581419
rs11117354
rs6881568
rs61748181
rs9878436
rs77732866
rs11699829
rs6669563
rs4530278
rs131797
rs117630647
rs80116508
rs6584579
rs76666449
rs2555104
rs113525195
rs45604339
rs4743037
rs8102497
rs150150565
rs6659669
rs11085072
rs61405042
rs11769630
rs34550383
rs66731853
rs450962
rs376641875
rs17803849
rs9940099
rs117407747
rs3891167
rs75664430
rs6751209
rs13230646
rs111527438
rs142730696
rs202034370
rs12932179
rs7221585
rs188918174
rs11212631
rs7099229
rs6587577
rs1023767
rs611646
rs1957937
A8
rs2276182
rs7164950
rs78491606
rs117034449
rs13062095
rs1291143
rs17677991
rs145114957
rs112394943
rs185174247
rs871134
rs1611236
rs2282764
rs7209057
rs76065543
rs80324517
rs2538745
rs3785074
rs9600019
rs6790988
rs139669835
rs55747751
rs6536702
rs429358
rs869785
rs1907702
rs111950327
rs28502153
rs6054257
rs3767952
rs12412214
rs11584821
rs5742915
rs1003322
rs2763979
rs79977579
rs6776756
rs7705526
rs182059586
rs4695407
rs16978028
rs4498805
rs41304832
rs116863223
rs76219171
rs56061761
rs11991877
rs117512405
rs59409453
rs8105767
rs4731541
rs142426306
rs932002
rs1332941
rs762679
rs56799554
rs4724
rs7790856
rs143190905
rs9419958
SNP
-0.025
0.000
0.025
0.050
Effect size (b)
